# A Novel AAF-SwinT Model for Automatic Recognition of Abnormal Goat Lung Sounds

**DOI:** 10.3390/ani16071021

**Published:** 2026-03-26

**Authors:** Shengli Kou, Decao Zhang, Jiadong Yu, Yanling Yin, Weizheng Shen, Qiutong Cen

**Affiliations:** 1 College of Electrical Engineering and Information, Northeast Agricultural University, Harbin 150030, China; koushengli@neau.edu.cn (S.K.); 17138755020@163.com (D.Z.); 18846906353@163.com (J.Y.); cya0502@yeah.net (Q.C.); 2 Network and Educational Technology Center, Harbin University of Commerce, Harbin 150028, China

**Keywords:** goat lung sounds, abnormal lung sound recognition, Swin Transformer, deep learning

## Abstract

In abnormal goat lung sound recognition, high inter-class similarity and large intra-class variability pose significant challenges. To address this issue and improve recognition performance, we propose a deep learning model, AAF-SwinT, based on an improved Swin Transformer. The model replaces the original Swin Transformer self-attention module with Axial Decomposed Attention (ADA), modeling the temporal and frequency axes separately and integrating attention weights to mitigate inter-class feature similarity. Adaptive Spatial Aggregation for Patch Merging (ASAP) is designed to emphasize key time-frequency regions, and a Frequency-Aware Multi-Layer Perceptron (FAM) is introduced to model features across different frequency bands, further enhancing the discriminative ability for abnormal lung sounds. Experiments on a self-constructed goat lung sound dataset demonstrate that AAF-SwinT achieves an accuracy of 88.21%, outperforming existing mainstream Transformer-based models by 2.68–5.98%. Ablation studies further confirm the effectiveness of each proposed module, improving the accuracy of baseline Swin Transformer model from 85.53% to 88.21%. These results indicate that the proposed approach exhibits strong robustness and practical potential for abnormal lung sound recognition in goats, providing technical support for early diagnosis and management of respiratory diseases in large-scale goat farming.

## Simple Summary

Respiratory diseases are common and frequently occur in goat farming. As key physiological signals reflecting the health status of the respiratory system, lung sounds play an important role in the diagnosis of respiratory diseases. Lung auscultation is a fundamental diagnostic method; however, it currently relies primarily on manual operation. The diagnostic outcomes are strongly influenced by the experience and skill level of veterinarians, resulting in subjectivity and limited accuracy. To achieve efficient and precise identification of goat respiratory diseases while reducing reliance on expert knowledge, this study collected goat lung sounds using a handheld contact-type electronic stethoscope and established a dedicated goat lung sound database. Using deep learning techniques, we developed an automatic recognition model for abnormal goat lung sounds. The results demonstrate that the proposed model achieves satisfactory recognition performance. This work contributes to the automatic monitoring and early warning of livestock respiratory health in practical applications and provides more efficient and intelligent support for health management in animal husbandry.

## 1. Introduction

Lung sounds are important physiological signals that reflect the health status of the respiratory system. Through auscultation, abnormal respiratory sounds such as rhonchi and pleural friction rub can be identified, providing important clinical evidence for the diagnosis of respiratory diseases [1,2,3]. However, traditional auscultation relies heavily on veterinarians’ experience, resulting in strong subjectivity, and its diagnostic accuracy is limited under complex noise conditions or in cases of mild pathological changes [4].

With the development of electronic stethoscopes and signal processing techniques, computer-aided lung sound recognition has achieved considerable progress in human healthcare [5,6]. However, studies focusing on ruminants, particularly goats, remain limited. In large-scale farming environments, respiratory diseases are characterized by rapid transmission and strong concealment, which may lead to significant economic losses. Therefore, developing automatic recognition methods for abnormal goat lung sounds is of great practical importance. At present, computer-aided diagnosis of lung sounds in ruminants is still in its early stage [7], while advances in human lung sound analysis have provided valuable references for applying related techniques to livestock scenarios.

Effective feature extraction is a key step in automatic lung sound recognition. In the early stage of human lung sound abnormality detection, time-domain features are mainly extracted, such as short-time energy, short-time zero-crossing rate, amplitude, envelope, mean, and standard deviation [8,9,10]. However, purely time-domain features are insufficient to characterize the non-stationary and nonlinear properties of lung sound signals. Consequently, frequency-domain analysis methods were gradually introduced. Demir et al. [11] converted lung sounds into spectrograms and short-time Fourier transform representations, achieving high classification accuracy. Jayalakshmy et al. [12] utilized Gammatone Cepstral Coefficients (GTCC) as acoustic features and performed classification based on these features, achieving promising results in lung sound classification. With further development, joint time–frequency representations became mainstream. Naqviet al. [13] combined time-domain, frequency-domain, and time–frequency-domain features with multiple classifiers for recognition. Mang et al. [14] performed classification by extracting spectrograms, Mel spectrograms, and CQT spectrograms from lung sound data. Gupta et al. [15] extracted gammatone spectrograms using a filter bank that simulates human auditory frequency analysis, enhancing abnormal lung sound representation.

In terms of modeling, early studies mainly relied on traditional machine learning methods such as support vector machines (SVM), k-nearest neighbors (k-NN), and random forests [16,17,18,19]. These approaches often suffer from low accuracy and instability, with limited generalization under complex noise conditions or high inter-class similarity. With the advent of deep learning, a new paradigm for lung sound diagnosis emerged. Unlike traditional methods that depend on handcrafted features, deep learning enables automatic feature learning through deep neural networks and has demonstrated superior performance across various tasks. Aykanat et al. [20] proposed a two-layer convolutional neural network (CNN) using MFCC features, outperforming traditional SVM models. In addition, CNNs have been employed to model lung sound spectrograms and achieved effective discrimination among multiple respiratory diseases [21,22,23,24]. Pham et al. [25] introduced a teacher–student CNN expert network based on Mel and gammatone spectrograms, achieving high recognition accuracy. Choi et al. [26] incorporated attention mechanisms into deep learning models, further improving classification accuracy by emphasizing critical features. To model temporal dependencies in lung sounds, recurrent neural networks (RNNs) were introduced. Kochetov et al. proposed an end-to-end RNN-based framework for abnormal lung sound detection [27,28]. Moreover, hybrid CNN-RNN/LSTM architectures were proposed to leverage both local feature extraction and sequential modeling capabilities [29,30,31]. In recent years, inspired by their success in computer vision, Transformer architectures and self-attention mechanisms have attracted increasing attention in lung sound analysis and agricultural artificial intelligence [32,33,34,35,36].

Building on the established research in human lung sound abnormality recognition, this study focuses on the intelligent identification of abnormal goat lung sounds. In livestock acoustic recognition, persistent challenges include heavily intertwined background noise, high inter-class similarity, and significant intra-class heterogeneity. While traditional CNNs are constrained by their local receptive fields, Transformer-based models break through these local spatial constraints to dynamically establish global spatio-temporal correlations. This capability allows the model to precisely focus on critical pathological acoustic frequency bands and temporal segments while effectively suppressing interference from non-stationary environmental noise, offering a robust solution for acoustic signal recognition in agricultural scenarios [37,38]. To this end, this study adopts the Swin Transformer, which possesses strong hierarchical local–global modeling capability, as the backbone network. On this basis, we further optimize the architecture from multiple perspectives, including multi-scale attention mechanisms, spatial feature aggregation strategies, and frequency-band adaptive modeling. Consequently, an improved AAF-SwinT model is proposed. The proposed model aims to enhance the network’s ability to capture and represent high-learning-difficulty abnormal sample features, thereby achieving high-accuracy and robust intelligent recognition of abnormal goat lung sounds in complex environments.

The main contributions of this work are summarized as follows:

(1) An axial decomposed attention (ADA) module is proposed to enhance lung sound feature representation by decomposing time–frequency attention modeling, thereby alleviating the feature similarity problem among different lung sound categories.
(2) An Adaptive Spatial Aggregation for Patch Merging (ASAP) is designed to adaptively weight and aggregate features from important regions, reducing intra-class feature variability caused by noise and individual differences.
(3) A Frequency-Aware Multi-Layer Perceptron (FAM) is proposed to improve the extraction of discriminative spectral information in lung sounds by applying differentiated feature transformation strategies across different frequency bands.

The remainder of this paper is structured as follows. Section 2 provides the materials and methods. Section 3 presents the experimental results, followed by the discussion of these results in Section 4. Finally, Section 5 provides the conclusions.

## 2. Materials and Methods

### 2.1. Data Collection

The lung sound data used in this study were collected from a large-scale dairy goat farm located in Tailai County, Qiqihar City, Heilongjiang Province, China. Data acquisition was conducted in April, which corresponds to a high-incidence period for respiratory diseases. The subjects included adult lactating goats that were either healthy or clinically diagnosed with respiratory diseases (e.g., pneumonia). Procedures involving animals were performed by licensed veterinarians with clinical experience based on established clinical diagnostic criteria for health status and respiratory diseases. Each goat’s physiological condition was assessed to ensure that the sampled individuals met the study requirements. During data acquisition, veterinarians were involved throughout the entire process, including determining auscultation sites, making preliminary assessments of abnormal respiratory sounds, and guiding standardized recording procedures. All recordings were obtained as part of routine health monitoring and clinical auscultation, without applying additional interventions or invasive procedures, thereby ensuring animal welfare and maintaining the natural authenticity of the data. To improve data quality, veterinarians assisted in applying appropriate physical restraint prior to recording so that the goats remained in a relatively stable and calm state. This approach helped enhance lung sound signal quality while minimizing stress to the animals. In addition, veterinarians provided preliminary clinical assessments based on on-site observations, which served as important reference information for subsequent data annotation and organization.

**Figure 1:**
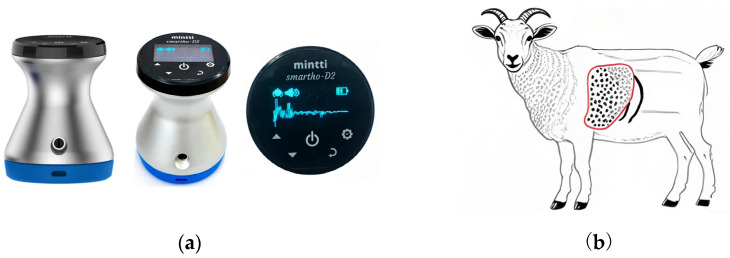
Data collection locations and auscultation equipment. (**a**) MinttiHealth Smartho-D2 Electronic Stethoscope. (**b**) The red area is the lung boundary area.

A MinttiHealth Smartho-D2 electronic stethoscope (Zhejiang Unoi Medical Technology Co., Ltd., Xianju, Zhejiang, China) was used to acquire lung auscultation signals, as shown in Figure 1a. The stethoscope operates within a frequency range of 20–2000 Hz and provides a signal amplification factor of 32. The recorded signals were wirelessly transmitted in real time to a mobile device via low-energy Bluetooth. During acquisition, the stethoscope was firmly placed on the lung boundary area of the goat, indicated by the red region in Figure 1b. The lung boundary region was anatomically defined as the area enclosed by the posterior edge of the scapula, the iliocostal muscle groove, the line extending anteriorly from the hip joint to the intersection with the eleventh rib, and the line extending posteriorly from the shoulder joint to the intersection with the eighth rib (extending downward to the fifth rib and upward to the iliocostal groove). The sampling rate of the electronic stethoscope was set to 8000 Hz with a resolution of 16 bits, using single-channel recording. Audio files were stored in “.wav” format and named according to the ear tag number of each goat. To comprehensively capture lung conditions, lung sounds from both the left and right sides of each goat were recorded.

### 2.2. Dataset Annotation

To ensure the accuracy and scientific reliability of the data annotation in this study, classification criteria for lung sound categories were established prior to the formal annotation process. These criteria were developed by comprehensively referencing veterinary clinical diagnostic textbooks and incorporating the opinions of licensed veterinarians with extensive clinical experience. All lung sound samples were annotated according to a mutually exclusive classification principle, meaning that each lung sound recording was assigned only one unique category label. The specific classification criteria are as follows:

(1) Normal: Lung auscultation sounds are clear, with inspiratory and expiratory phases alternating smoothly and rhythmically, and no identifiable abnormal adventitious sounds. The respiratory rate remains within the normal resting range.
(2) Rhonchi: Continuous, low-pitched abnormal respiratory sounds resembling snoring or groaning can be heard during auscultation.
(3) Tachypnea: The respiratory rate is significantly higher than the normal physiological range for goats (typically 12–30 breaths per minute), manifested as a noticeably accelerated breathing rhythm, which may not necessarily be accompanied by obvious abnormal adventitious sounds. This category is primarily determined based on abnormal respiratory rate.
(4) Noise: The audio contains significant interference from non-lung sound signals, such as friction sounds caused by animal movement, stethoscope contact noise, environmental background noise, or other non-respiratory acoustic signals, which prevent accurate reflection of true lung sound characteristics.

**Figure 2:**
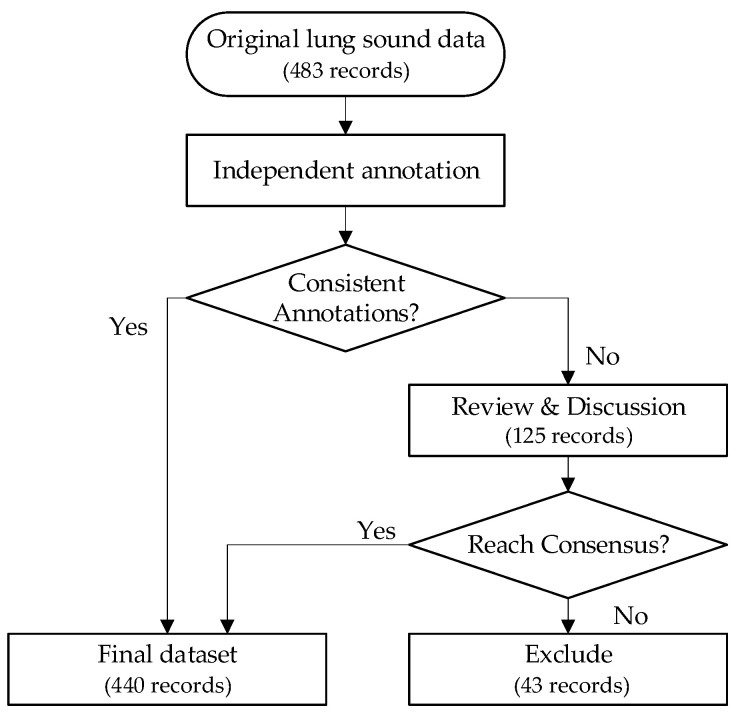
Flowchart of Data Annotation.

Based on the above classification criteria, the dataset annotation was jointly conducted by three experts with extensive clinical diagnostic experience, including a university professor specializing in clinical veterinary medicine, a researcher from an institute of animal husbandry and veterinary science, and a licensed farm veterinarian who was also involved in data collection and clinical evaluation. The annotation process was carried out using Cool Edit Pro 2.1 audio processing software. During annotation, the three experts first reviewed individual clinical information recorded by the attending veterinarian during data acquisition (e.g., body temperature changes, coughing symptoms, nasal discharge, and other respiratory-related clinical signs). Based on these references, each expert independently listened to all lung sound recordings and completed the initial labeling. For samples with inconsistent annotations, collective discussions and re-evaluations were conducted. Final decisions were made through consensus by integrating acoustic characteristics of lung sounds with clinical experience. Samples for which agreement could not be reached after repeated discussions were excluded to ensure the accuracy, scientific validity, and clinical reliability of the dataset labels. The annotation process is illustrated in Figure 2. The data labeling format followed the annotation protocol of the publicly available ICBHI 2017 dataset [39], whereby each “.wav” lung sound audio file corresponded to a text label file with the same filename in “.txt” format to record the classification label. This standardized organization enhances data consistency and reproducibility. By annotating and screening 483 raw recordings, a total of 440 valid lung sound recordings from 104 goats were ultimately obtained. The final structure of the dataset and the labels for each category are shown in Table 1.

### 2.3. Data Preprocessing and Feature Extraction

**Figure 3:**
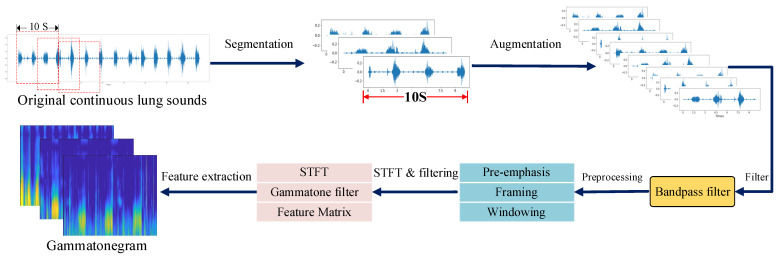
Data preprocessing and feature extraction process.

Before classification, the annotated lung sound data were subjected to preprocessing and feature extraction. The overall processing pipeline is illustrated in Figure 3.

#### 2.3.1. Data Segmentation

The duration of raw audio recordings varied across samples. In clinical practice, veterinarians can typically make a preliminary diagnosis based on lung sounds covering 3–5 respiratory cycles. Therefore, in this study, each audio recording was segmented into 10 s samples using a 50% overlap strategy. After segmentation, a dataset consisting of 4173 samples was constructed to support subsequent model training and analysis. The number of samples across different lung sound categories is presented in Table 2.

#### 2.3.2. Data Augmentation

Based on the strict physiological and pathological semantic constraints of lung sound signals, unlike conventional speech, the underlying acoustic properties of lung sounds directly reflect specific health states. Physically altering the raw waveform can easily disrupt the time–frequency microstructure of key pathological features, introducing uncontrollable semantic noise. In this study, the Synthetic Minority Over-sampling Technique (SMOTE) was employed to perform data augmentation through interpolation of feature vectors in the abstract feature space rather than manipulating raw audio. The number of samples for each category was expanded to match the size of the largest class (Normal lung sounds, 1824 recordings) to achieve a balanced dataset. After data augmentation, a total of 7296 ten-second data segments across all categories were obtained.

#### 2.3.3. Bandpass Filtering

During data acquisition, lung sound recordings are inevitably contaminated by various types of noise. To reduce the influence of noise on model performance, a bandpass filter was applied. Since lung sound energy is mainly distributed within the frequency range of 50–2000 Hz, a fourth-order Butterworth bandpass filter with a lower cutoff frequency of 50 Hz and an upper cutoff frequency of 2000 Hz was applied to remove out-of-band noise components.

#### 2.3.4. Feature Extraction

In this study, lung sound signals were transformed into gammatone spectrograms, which served as the input to the classification model for deep feature extraction. The gammatone filter bank simulates the frequency analysis characteristics of the human auditory system. Its impulse response function can be expressed as

(1) $$g\left(t\right)=at^{n-1}e^{-2\pi bERB\left(f_{c}\right)t}\cos\left(2\pi f_{c}t+\phi \right)$$

where *a* is the filter gain, *n* is the filter order, *b* is the decay factor (typically set to 1.019), $ERB\left(f_{c}\right)$ represents the equivalent rectangular bandwidth, $f_{c}$ denotes the center frequency, and ϕ is the phase. Compared with conventional time–frequency analysis methods such as short-time Fourier transform and wavelet transform, gammatone spectrograms can more accurately characterize the fine-grained time–frequency details of respiratory sounds and preserve physiological information embedded in lung sounds. They offer superior representation capability for both low- and high-frequency variations, making them well suited for lung sound analysis.

### 2.4. Data Analysis

**Figure 4:**
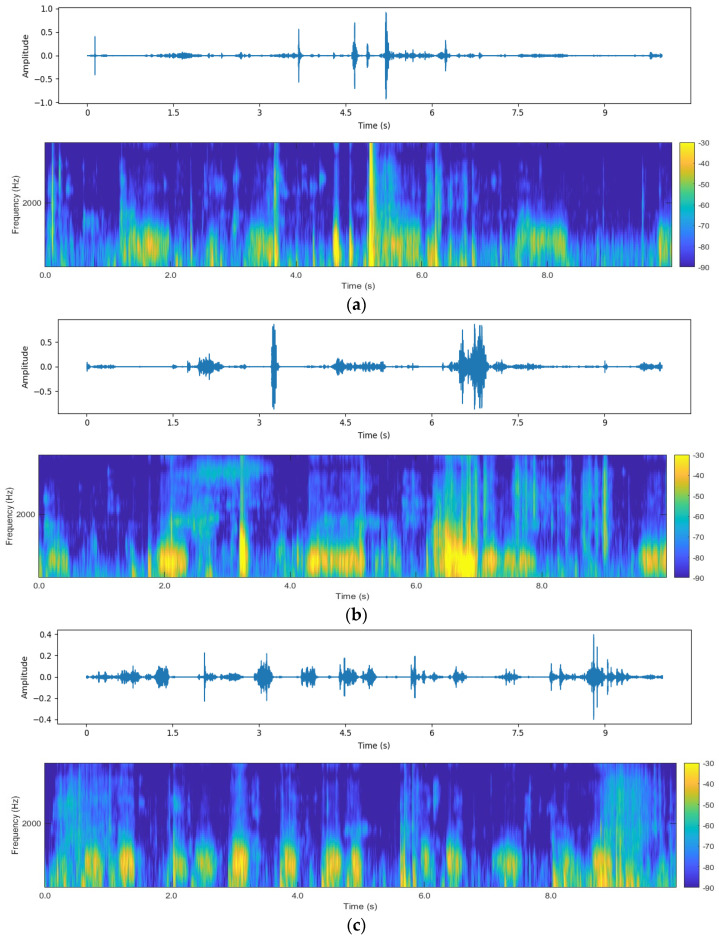
Waveforms and spectrograms of various types of sounds. (**a**) Waveform and spectrogram of normal lung sounds. (**b**) Waveform and spectrogram of rhonchi. (**c**) Waveform and spectrogram of tachypneic.

Figure 4 presents representative examples of time-domain waveforms and corresponding time–frequency spectrograms for three typical lung sound categories over a 10 s duration: normal lung sounds, rhonchi, and tachypneic lung sounds. As observed, all three types exhibit clear periodic fluctuations in the time domain corresponding to inhalation and exhalation phases, with energy primarily concentrated during the inhalation stage. From a time–frequency perspective, the three categories show substantial similarity in spectral distribution, with most energy located in the low- to mid-frequency range. The primary differences lie in spectral bandwidth, energy intensity, and respiratory rhythm. Compared with normal lung sounds, rhonchi exhibit stronger energy intensity, while tachypneic lung sounds display denser spectral stripes and faster respiratory rates.

**Figure 5:**
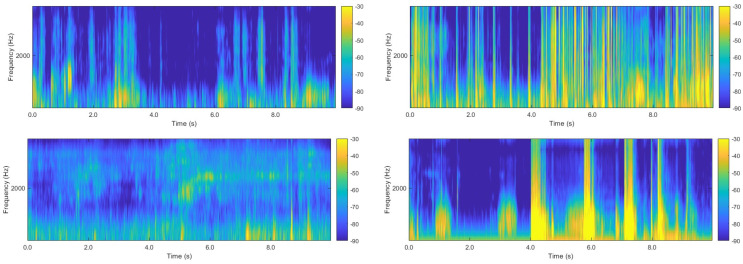
Display of noise spectrograms.

To better reflect real auscultation scenarios, noise was intentionally included as an independent classification category. Representative noise spectrograms are shown in Figure 5. Noise signals exhibit significant diversity in both time and frequency domains. Some noise samples consist of high-amplitude random noise that severely masks effective lung sounds, while others are dominated by environmental noise with minimal lung sound information. Notably, certain noise samples exhibit spectral patterns similar to low-quality lung sounds (e.g., weak rhonchi or normal lung sounds with low signal-to-noise ratios), with energy concentrated in low-frequency regions. This similarity reduces inter-class separability and increases the difficulty of subsequent classification tasks.

### 2.5. AAF-SwinT Model

#### 2.5.1. Overall Architecture

**Figure 6:**
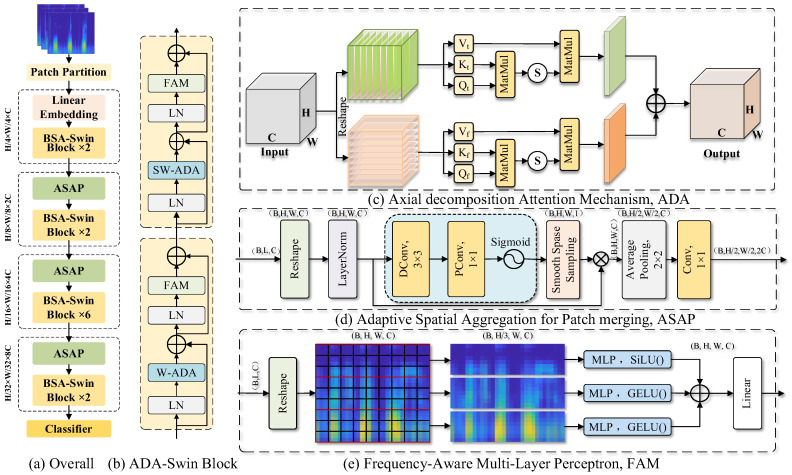
Overall architecture and module structures. (**a**) Overall model architecture. (**b**) ADA-Swin block structure. (**c**) ADA-Swin block structure. (**d**) ASAP block structure. (**e**) FAM block structure.

In goat lung sound dataset, feature similarity between certain rhonchi and normal lung sounds, intra-class heterogeneity, and the presence of low-quality samples caused by complex noise environments pose significant challenges to classification models, leading to poor generalization and low recognition accuracy. To address these challenges, this study proposes a novel lung sound classification model termed AAF-SwinT, as illustrated in Figure 6a. The model adopts Swin Transformer as the baseline architecture to leverage its hierarchical feature extraction capability for capturing multi-scale acoustic features. Specifically, the original window-based self-attention mechanism is replaced with an ADA module; the standard Patch Merging operation is optimized into an ASAP to preserve critical features; and the conventional MLP is replaced with a FAM to better accommodate the characteristics of abnormal goat lung sound recognition. These improvements collectively enhance the model’s accuracy and robustness.

#### 2.5.2. Axial Decomposed Attention Mechanism

Lung sound signals, as a type of acoustic signal, exhibit characteristic distribution patterns in both the time and frequency domains. During feature extraction, jointly modeling the relationships between temporal and frequency domain features in the feature map enables the model to achieve improved performance. However, when Swin Transformer employs the conventional multi-head self-attention mechanism to compute attention weights, it performs global attention computation along a single dimension, thereby overlooking the distinct characteristics inherent to the temporal and frequency dimensions. To more effectively capture these properties and to alleviate the adverse effects of high inter-class similarity and large intra-class variability on lung sound recognition performance, this study proposes an ADA mechanism inspired by time–frequency attention mechanism, as illustrated in Figure 6c. The proposed module preserves the fundamental principle of multi-head self-attention while decomposing window-level features along two orthogonal axes, namely the temporal and frequency axes. Attention computation is then performed independently along each dimension to extract intra-dimensional feature dependencies. Finally, the outputs of the temporal and frequency branches are aggregated to emphasize salient time–frequency patterns.

For the input feature tensor $X\in R^{B\times H\times W\times C}$, *B* denotes the batch size, *H* and *W* represent the frequency and time dimensions, respectively, and *C* denotes the feature dimension. The ADA first applies a shared linear projection to the input features to generate the query (*Q*), key (*K*), and value (*V*) matrices required for attention computation.

(2) $$Q,K,V=Linear(X)\in R^{B\times H\times W\times C}$$

To enable attention computation along the temporal axis and the frequency axis, *Q, K*, and *V* are required to undergo dimensional rearrangement. Specifically, they are reshaped into a multi-head format and their dimension order is adjusted accordingly. The operation functions F and G are defined for the temporal branch and the frequency branch as follows:

(3) $$F\left(M\right)=Reshap\left(M\right)\cdot permute\left(0,1,3,2,4\right)$$

(4) $$G\left(M\right)=Reshap\left(M\right)\cdot permute\left(0,2,3,1,4\right)$$

where *M* represents the input matrix. Through the above operations, the query, key, and value matrices in the temporal branch are obtained as $Q_{t}=F\left(Q\right);K_{t}=F\left(K\right);V_{t}=F(V)$. Similarly, for the frequency branch, the corresponding matrices are given by $K_{f}=G(K)$ and $V_{f}=G(V)$. Attention computation is then performed independently along the two dimensions:

(5) $${Score}_{t}=\mathrm{Softmax}\left(\frac{Q_{t}K_{t}^{T}}{\sqrt{d_{k}}}\right)V_{t}$$

(6) $${Score}_{f}=\mathrm{Softmax}\left(\frac{Q_{f}K_{f}^{T}}{\sqrt{d_{k}}}\right)V_{f}$$

where $d_{k}$ denotes the dimensionality of the key vectors. The Softmax function is used to normalize the attention weights so that their values fall within the range [0, 1], ${Score}_{t}$ and ${Score}_{f}$ represent the attention outputs of the temporal branch and the frequency branch, respectively. Finally, the outputs of the temporal and frequency branches are concatenated and projected through a linear transformation to obtain the final output Output1*:*

(7) $$Output1={Linear}_{2C\to C}(Concat({Score}_{t},{Score}_{f}))\in R^{B\times H\times W\times C}$$

#### 2.5.3. Adaptive Spatial Aggregation for Patch Merging

In the conventional Swin Transformer, downsampling is performed through the Patch Merging module using a fixed 2 × 2 grid, where all regions are treated uniformly. This strategy ignores the differences in importance among features from different spatial regions. However, in lung sound spectrograms, different frequency bands or time segments may contain unequal amounts of discriminative information. During real-world data acquisition, lung sounds belonging to the same class often exhibit substantial variations across different individuals, respiratory phases, and noise conditions, leading to unstable or redundant features and consequently exacerbating intra-class feature variability. To address this issue, we design an ASAP module, which adaptively weights different spatial regions of the feature map to enhance stable and discriminative regions while suppressing regions that are highly affected by noise or individual differences. In this way, ASAP effectively reduces spatial feature fluctuations within the same class and alleviates the problem of excessive intra-class feature variation, as illustrated in Figure 6d.

First, the input feature $X\in R^{B\times L\times C}$, where L denotes the sequence length, is reshaped into the spatial format (*B*, *C*, *H*, *W*) and normalized. Subsequently, a depthwise separable convolution followed by a channel reduction operation is employed to generate a salience feature map. The depthwise separable convolution consists of a depthwise convolution and a pointwise convolution. The depthwise convolution performs independent convolution operations on each input channel to effectively extract local spatial features, while the pointwise convolution linearly combines multi-channel feature maps to achieve channel reduction. The resulting salience feature map is computed as follows:

(8) $$Salience=\sigma \left({Conv}_{1\times 1}\left({\mathrm{DepthwiseConv}}_{3\times 3}\left(X\right)\right)\right)\in R^{B\times 1\times H\times W}$$

where σ denotes the Sigmoid activation function, which maps the output values to the range [0, 1]. Next, smooth sparse sampling is performed on the salience feature map. A learnable threshold α is applied to filter the salience map and generate a smooth sampling mask.

(9) $$Mask=\sigma \left(10\cdot \left(Salience-\alpha \right)\right)\in R^{B\times 1\times H\times W}$$

Here, the coefficient 10 is introduced to enhance the nonlinearity of the Sigmoid function, allowing the mask to exhibit a steeper transition when the salience score is close to the threshold α, thereby enabling more effective selection of important features. The generated mask is then applied to the input feature map through element-wise multiplication to obtain the weighted feature map $F_{w}$:

(10) $$F_{w}=X\cdot Mask\in R^{B\times C\times H\times W}$$

Subsequently, a 2 × 2 average pooling operation is applied to the weighted feature map to perform spatial downsampling, resulting in the pooled feature map $F_{p}$. Finally, a 1 × 1 convolution is used to expand the channel dimension from *C* to 2*C*, yielding the final output feature *Output*2, which can be expressed as

(11) $$F_{p}={AvgPool}_{2\times 2}\left(F_{w}\right)\in R^{B\times C\times \frac{H}{2}\times \frac{W}{2}}$$

(12) $$Output2={Conv}_{1\times 1}\left(F_{p}\right)\in R^{B\times 2C\times \frac{H}{2}\times \frac{W}{2}}$$

#### 2.5.4. Frequency-Aware Multi-Layer Perceptron

Lung sound spectrogram features exhibit distinct information distributions across different frequency bands. Normal lung sounds, rhonchi, and tachypneic lung sounds are mainly concentrated in the low-frequency range, while the mid-frequency range contains relatively limited information. Moreover, the overall energy intensity of rhonchi and tachypnea in this range is generally higher than that of normal lung sounds. In contrast, high-frequency bands mainly contain abnormal sounds and various types of noises, such as environmental noises and friction sounds. Conventional multi-layered perceptron (MLP) applies the same processing strategy to all frequency bands, ignoring band-specific characteristics and failing to fully exploit the unique information embedded in different frequency regions. Therefore, to better capture frequency-dependent features, this study proposes a FAM that explicitly leverages the distinctive characteristics of different frequency bands, as illustrated in Figure 6e.

First, the input feature $X\in R^{B\times L\times C}$ is reshaped into a spatial format $X\in R^{B\times H\times W\times C}$. Subsequently, the feature map is split along the frequency axis into three sub-bands, namely low, mid, and high frequency bands, denoted as $X_{low}$, $X_{mid}$, and $X_{high}$, respectively:

(13) $$X_{low},X_{mid},X_{high}=Split\left(X,\left[\frac{H}{3},\frac{H}{3},H-2\times \left(\frac{H}{3}\right)\right],dim=1\right)$$

Audio information from different frequency bands exhibits varying levels of complexity. Low-frequency components change slowly and have relatively low complexity; therefore, a small-capacity network with the GELU activation function is employed to reduce computational cost and mitigate overfitting. High-frequency components change rapidly and contain more complex details; thus, a larger-capacity network combined with the SiLU activation function is adopted to capture rich spectral details. The mid-frequency band exhibits moderate complexity, and a standard-capacity network with a conventional activation function is used to balance feature extraction capability and model complexity. This design enables the model to adaptively match the characteristics of different frequency bands when processing lung sound spectrograms. The operations can be formulated as follows.

(14) $$\left\{\begin{array}{c} {Out}_{low}={Linear}_{\frac{C}{2}\to C}\left(GELU\left({Linear}_{C\to \frac{C}{2}}\left(X_{low}\right)\right)\right)\\ {Out}_{mid}={Linear}_{C\to C}\left(GELU\left({Linear}_{C\to C}\left(X_{mid}\right)\right)\right)\\ {Out}_{high}={Linear}_{2C\to C}\left(SiLU\left({Linear}_{C\to 2C}\left(X_{high}\right)\right)\right) \end{array}\right.$$

Finally, the outputs from the three frequency bands are concatenated along the frequency dimension and processed by a linear layer to obtain the final output feature:

(15) $$Output3={Linear}_{C\to C}\left(Concate\left({Out}_{low},{Out}_{mid},{Out}_{high},dim=1\right)\right)\in R^{B\times H\times W\times C}$$

## 3. Results

### 3.1. Model Evaluation and Evaluation Metrics

**Figure 7:**
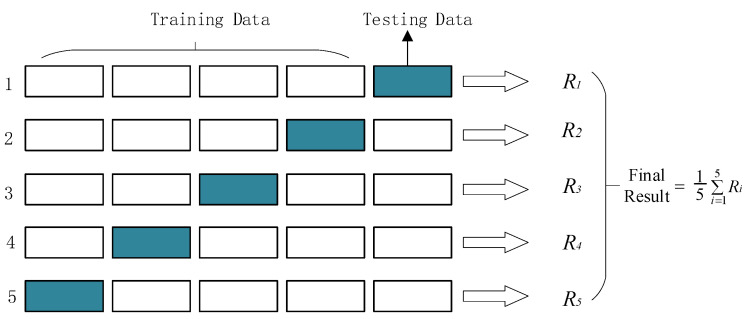
Schematic diagram of 5-fold cross-validation.

Due to the limited amount of data obtained in this study, it is difficult to divide the dataset ideally into a “training set” for learning data characteristics, a “validation set” for tuning the hyperparameters of the learning model, and a “test set” for evaluating model performance. Therefore, this study employs k-fold cross-validation to evaluate the model [40]. In this study, the data are randomly divided into five roughly equal folds. In each iteration of the cross-validation process, one fold is used to test the learning algorithm, while the remaining folds are used for training, as shown in Figure 7. This process is repeated five times so that all folds are used in the testing phase, and the average performance across the test folds is computed as an unbiased estimate of the overall performance of the algorithm.

To comprehensively evaluate the performance of the abnormal lung sound recognition model, four metrics were adopted: Accuracy, Sensitivity (*Se*), Specificity (*Sp*), and *Score*. The mathematical definitions of these metrics are given in Equations (16)–(19):

(16) $$Accuracy=\frac{Nl_{nl}+W_{w}+T_{t}+Ne_{ne}}{Nl+R+T+Ne}$$

(17) $$Se=\frac{W_{w}+T_{t}}{W+T}$$

(18) $$Sp=\frac{{Nl}_{nl}}{Nl}$$

(19) $$Score=\frac{Sp+Se}{2}$$

where *Nl*, *W*, *T*, and *Ne* denote the total numbers of Normal lung sound, Rhonchi, Tachypnea, and Noise respectively, and *Nl_nl_*, *W_w_*, *T_t_*, and *Ne_ne_* represent the correctly classified samples for each category. Accuracy reflects the proportion of correctly classified samples across all classes. Sensitivity measures the model’s ability to correctly identify abnormal lung sounds (rhonchi and tachypnea), while specificity evaluates the correct recognition of normal lung sounds. The Score metric provides a balanced assessment of sensitivity and specificity.

### 3.2. Parameter Settings

All experiments were implemented on a workstation equipped with an Intel Core i7-13620H CPU (4.0 GHz, manufactured by Intel Corporation, Santa Clara, CA, USA), 16 GB RAM, and an NVIDIA GeForce RTX 4060 Laptop GPU (8 GB). The experiments were implemented using PyCharm 2022, with Python 3.8 and the PyTorch 2.0.0 deep learning framework. The hyperparameter settings are summarized in Table 3. The learning rate was set to 0.0001, the batch size to 32, and the number of training epochs to 50. The Adam optimizer was employed, and the cross-entropy loss function was used for optimization. To ensure reliable performance evaluation, five-fold cross-validation was adopted.

### 3.3. Comparison of Different Segmentation Durations

To quantify the influence of audio segmentation duration on model performance, a controlled experiment was designed in which all preprocessing steps were kept identical while only the segmentation length was varied from 6 s to 12 s. The experimental results are presented in Table 3. As shown, classification accuracy initially increases and then decreases as segmentation duration increases. When the segment length increases from 6 s to 10 s, model performance improves significantly, with accuracy rising from 82.56% to 87.56%. This indicates that longer segments help capture more complete respiratory cycles and abnormal lung sound characteristics. However, when the segment duration exceeds 10 s, performance no longer improves and instead slightly declines. This phenomenon is mainly attributed to the reduction in available training samples caused by longer segmentation lengths, which negatively affects model generalization. Consequently, 10 s was selected as the optimal segmentation duration in this study.

### 3.4. Data Augmentation Analysis

**Figure 8:**
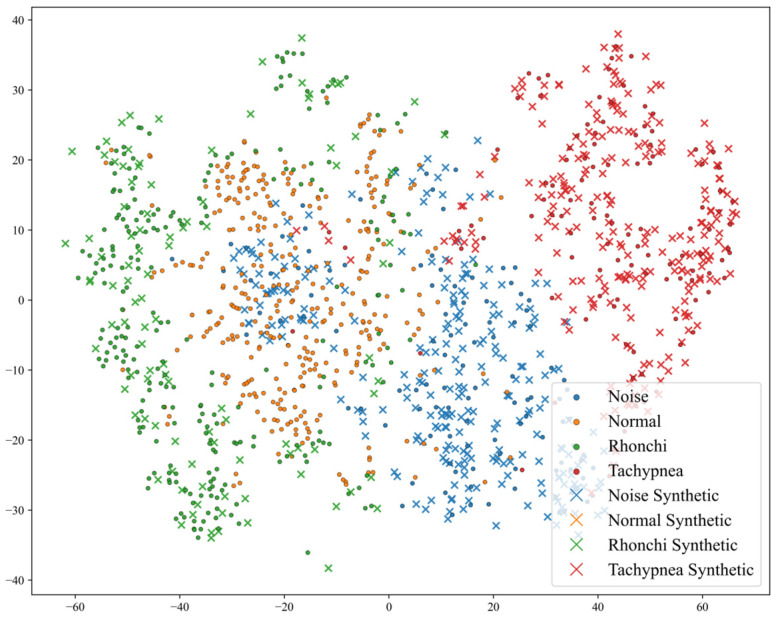
t-SNE Visualization of Feature Distribution After Data Augmentation.

As shown in Figure 8, the synthetic samples generated by SMOTE are fully distributed within the feature space defined by the real minority-class samples and effectively fill the regions of originally lower density within that class. This indicates that the augmentation process does not compromise the original inter-class separability. The visualization demonstrates that the interpolation-based synthesis performed by SMOTE in the feature space can generate synthetic samples highly consistent with the true pathological distribution, without introducing abnormal features that deviate from physiological semantics.

To further verify the effectiveness of the data augmentation, we compared the model performance before and after augmentation. As shown in Table 4, on the training set augmented with SMOTE, the model achieved notable improvements across all performance metrics on the independent test set, effectively confirming the validity of the data augmentation.

### 3.5. Comparison of Different Features

To validate the effectiveness of Gammatonegram in the abnormal goat lung sound recognition task, this study designed a comparative experiment. Gammatonegram was compared with commonly used audio features, including MFCC, Constant-Q transform (CQT) spectrograms, short-time Fourier transform (STFT) spectrograms, and Mel-spectrograms. All features were evaluated under the same AAF-SwinT model structure to ensure fairness and comparability of the experimental results. The results are shown in Table 5. As can be seen, among all compared features, Gammatonegram achieved the best performance across all evaluation metrics, indicating that it possesses stronger representational capability for the abnormal goat lung sound recognition task.

Compared to traditional acoustic features, the Gammatone filterbank better simulates the frequency selectivity characteristics of the human auditory system, offering higher resolution in the low-frequency range and more effectively capturing subtle variations in lung sound signals. Therefore, in the goat lung sound classification task, Gammatonegram provides more discriminative time–frequency feature representations, enhancing the model’s ability to recognize abnormal lung sounds. These experimental results provide strong support for selecting Gammatonegram as the feature extraction method in this study.

### 3.6. Comparison with Different Models

To verify the classification performance of the proposed AAF-SwinT model on the goat lung sound dataset, it was compared with several Transformer-based models, including Swin Transformer [41], ViT, MobileViT [42], DaViT, and DeiT. The comparison results are summarized in Table 6. Overall, the proposed AAF-SwinT model achieves the best performance across all evaluation metrics. Specifically, it attains an Accuracy of 88.21%, significantly outperforming all comparison models. Compared with the baseline Swin Transformer, AAF-SwinT improves Accuracy by 2.68% and Score by 1.81%. Compared with other Transformer-based architectures, AAF-SwinT also demonstrates superior discriminative capability, achieving Accuracy improvements of 2.96% over DaViT, 4.92% over ViT, and 5.98% over DeiT. Moreover, AAF-SwinT achieves a Sensitivity of 86.99% and a Specificity of 89.14%, indicating a well-balanced classification performance. Compared with the lightweight MobileViT model, AAF-SwinT improves Accuracy by 4.76% and Score by 3.66%.

**Figure 9:**
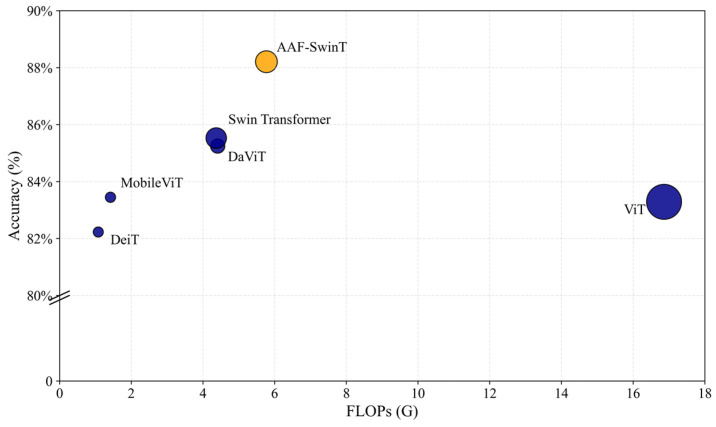
FLOPs of different models.

Figure 9 illustrates the trade-off between classification accuracy and computational cost (FLOPs), where the size of each circle represents the number of parameters. As shown, AAF-SwinT achieves the highest accuracy while maintaining moderate computational complexity and parameter size, indicating a favorable balance between performance and efficiency.

### 3.7. Evaluation of Different Attention Mechanisms

To comprehensively evaluate the effectiveness of the proposed ADA module in abnormal goat lung sound recognition, a series of comparative experiments were conducted using different attention mechanisms.

#### 3.7.1. Impact of Attention Computation on Different Dimensions

To investigate the impact of attention computation along different dimensions, four attention strategies were compared under identical experimental settings: standard multi-head self-attention without dimension distinction (MSA), temporal-only attention (TA), frequency-only attention (FA), and the proposed ADA. The results are shown in Table 7. Compared with standard MSA, applying attention solely along the temporal or frequency dimension leads to moderate performance improvements, indicating that strengthening intra-dimensional feature correlations is beneficial for lung sound representation. Among all strategies, the proposed ADA achieves the best performance, with an Accuracy of 88.21%. This demonstrates that joint modeling of temporal and frequency dimensions enables more effective exploitation of time–frequency characteristics and significantly reduces feature confusion between lung sound categories.

#### 3.7.2. Comparison with Other Attention Modules

**Figure 10:**
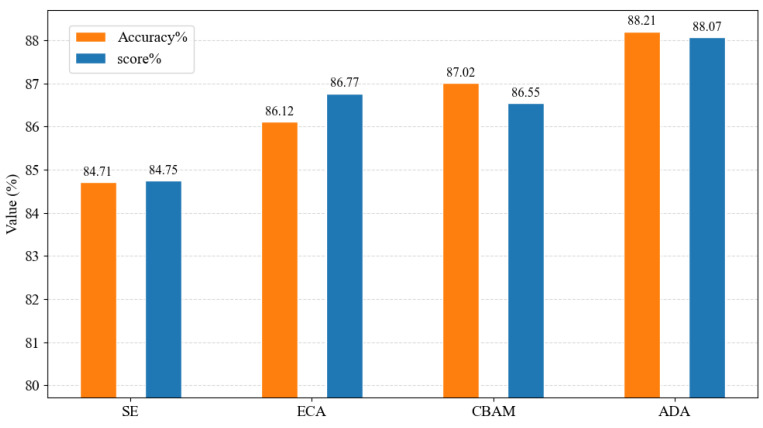
Comparison of different attention mechanisms.

In the standard Swin Transformer, attention is implemented using window-based multi-head self-attention. To validate the suitability of self-attention mechanisms for lung sound recognition, the proposed approach was compared with commonly used attention modules, including SE, ECA, and CBAM. As shown in Figure 10, compared with the SE and ECA attention mechanisms, the self-attention-based approach achieves superior performance in terms of both Accuracy and Score. Although CBAM further enhances classification performance by jointly modeling channel and spatial attention, its overall performance remains inferior to the proposed ADA module. These results confirm that self-attention mechanisms are particularly effective for modeling time–frequency dependencies in lung sound signals, justifying their continued use within the Swin Transformer framework.

### 3.8. Ablation Studies

To further validate the effectiveness of each proposed module, ablation experiments were conducted using Swin Transformer as the baseline model. The experimental results are summarized in Table 8. The baseline Swin Transformer achieves an Accuracy of 85.53%, Sensitivity of 84.76%, Specificity of 87.77%, and a Score of 86.26%. Introducing the ADA module improves Accuracy and Score by 0.42% and 0.81%, respectively, demonstrating that ADA enhances discriminative performance by strengthening time–frequency interactions, albeit at the cost of increased computational complexity. When only the ASAP is introduced, Accuracy and Score improve by 0.56% and 0.53%, respectively, while FLOPs decrease from 4.37 G to 4.25 G. This improvement results from the use of depthwise separable convolutions, which reduce computational cost while adaptively emphasizing salient spatial features. Combining ADA and ASAP further improves performance, with Accuracy and Score increasing by 1.11% and 1.15%, respectively, indicating strong complementarity between the two modules. Adding the FAM module yields the best overall performance, with Accuracy and Score improvements of 2.68% and 1.81%, respectively. This gain is attributed to the enhanced modeling of frequency-band-specific characteristics, although it also leads to increased computational complexity.

**Figure 11:**
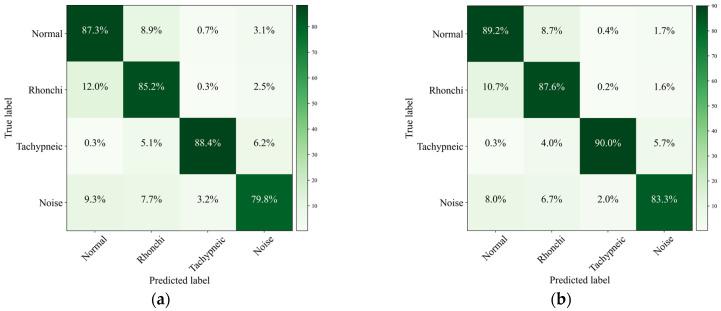
Confusion matrix of lung sound classification. (**a**) Swin Transformer. (**b**) AAF-SwinT.

Figure 11 presents the confusion matrices of Swin Transformer and AAF-SwinT. Compared with the baseline, AAF-SwinT achieves improved classification performance across all categories. Notably, the misclassification rate of rhonchi as normal lung sounds decreases from 12.0% to 10.7%, indicating enhanced inter-class separability. In addition, the proportion of samples misclassified as noise is reduced for all categories, demonstrating improved robustness against noise interference.

## 4. Discussion

This study focuses on abnormal goat lung sound recognition and proposes an improved Swin Transformer-based model, termed AAF-SwinT, for deep feature extraction and classification of goat lung sounds. Experimental analysis verifies the effectiveness of AAF-SwinT. In Section 3.6, we compared the proposed model with multiple mainstream Transformer models, and the results demonstrate that AAF-SwinT outperforms the comparison models on the goat lung sound dataset. It achieves an Accuracy of 88.21%, representing a 2.68% improvement over the baseline Swin Transformer, while maintaining a favorable balance between Sensitivity (86.99%) and Specificity (89.14%). Swin Transformer, as a classical hierarchical vision Transformer model, has demonstrated strong capability in global feature modeling in previous human lung sound recognition studies [43]. This study further verifies the extensibility of Transformer-based models to livestock lung sound recognition tasks.

Regarding attention mechanisms, experimental results in Section 3.7 show that general-purpose attention modules such as SE, ECA, and CBAM provide limited performance improvement. Similar observations have been reported in related studies, where generic attention mechanisms fail to effectively capture task-specific structural dependencies in acoustic signals [44]. This is mainly because these methods focus on global channel or spatial dimensions without explicitly modeling the interaction between temporal and frequency dimensions inherent in lung sound signals. In contrast, the proposed ADA module independently computes attention along temporal and frequency axes and makes a fusion, effectively strengthening time–frequency correlations and reducing feature confusion between rhonchi and normal lung sounds. The misclassification rate of rhonchi as normal sounds is reduced from 12.0% to 10.7%.

In this study, the introduction of the FAM further enhances the model’s ability to represent frequency-dependent information, leading to an additional 1.57% improvement in accuracy over the baseline model [41]. Previous studies have shown that lung sound signals exhibit significant differences in information distribution across frequency bands [45]. Normal lung sounds, rhonchi, and tachypnea are mainly concentrated in low- and mid-frequency ranges, whereas high-frequency regions often contain abnormal sounds or noise. By modeling different frequency bands with tailored network capacities, FAM enables the model to focus on diagnostically relevant spectral regions while suppressing irrelevant interference.

The performance improvement of AAF-SwinT is accompanied by a moderate increase in computational complexity. Compared with the baseline model [46], the floating-point operations increase by 29.06%, and the number of parameters increases by 2.68 times. This trade-off between performance and complexity is acceptable in complex livestock farming environments. Lightweight Transformer models such as MobileViT [42] have been widely used in human lung sound recognition to balance performance and computational efficiency, and the findings of this study provide useful references for the lightweight optimization of goat lung sound recognition models. It is worth noting that there are currently no publicly available goat lung sound datasets or related recognition studies. This work represents an initial attempt to apply Transformer-based models to goat lung sound recognition, thereby enriching research in abnormal lung sound analysis for ruminants.

Although this study has achieved significant results, several limitations still remain. First, the dataset was collected from one goat farm, and the generalization ability of the model across different environments and animal types requires further validation. Second, although data augmentation was applied, the relatively small dataset size may still influence model training stability. Finally, compared with lightweight models, AAF-SwinT still has room for optimization in terms of computational efficiency [47].

At the engineering application level, the development of an end-to-end intelligent auscultation system needs to consider the efficiency of the overall processing pipeline. System latency is determined collectively by the processing time of multiple modules, including preprocessing, feature extraction, and model inference, and any stage’s computational load directly affects the overall system performance. In particular, during the preprocessing and feature extraction phases, multi-step filtering and spectral transformation operations involve a certain amount of computational costs. Relevant studies have indicated that these processes are among the main computational burdens in resource-constrained embedded systems [48].

Future efforts will focus on diversifying the dataset by incorporating lung sound recordings from various breeds, geographical locations, and management systems, specifically including dairy goats across different age groups and parities to improve model robustness. To facilitate deployment, we will continue optimizing the model through lightweight techniques, such as pruning and knowledge distillation, aiming to mitigate computational costs without compromising performance. Furthermore, advanced preprocessing algorithms and hardware acceleration strategies will be explored to enhance real-time efficiency. The ultimate goal is to develop a comprehensive end-to-end diagnostic platform that synergizes electronic stethoscopes, real-time transmission, and optimized deep learning models, enabling fully automated “acquisition-to-diagnosis” functionality.

## 5. Conclusions

For the task of abnormal goat lung sound recognition, this study proposes an improved Swin Transformer-based model, termed AAF-SwinT. By incorporating an axial decomposed attention module, an adaptive spatial aggregation module, and a frequency-aware module, the proposed model is able to more effectively capture the correlations of lung sound signals across temporal and frequency dimensions, highlight salient time–frequency information, and adapt to the characteristic differences among frequency bands, thereby significantly enhancing the recognition capability for abnormal lung sounds. Experimental results on a self-constructed goat lung sound dataset demonstrate that the proposed model achieves an Accuracy of 88.21% and a Score of 88.07%, significantly outperforming several mainstream models. Further comparative experiments on attention mechanisms and comprehensive ablation studies verify the rationality and effectiveness of each proposed module. By jointly considering recognition performance and computational complexity, AAF-SwinT demonstrates strong practical applicability while maintaining high recognition accuracy. The proposed method provides a feasible and effective technical solution for intelligent identification of goat lung diseases and offers valuable support for livestock health monitoring and precision farming. In future work, we will focus on further improving model performance, expanding the scale and diversity of datasets, and exploring the applicability of the proposed method to other livestock species.

## Figures and Tables

**Table 1:** Composition of the goat lung sound dataset.

Types of Lung Sound Data	Normal	Rhonchi	Tachypnea	Noise
Label	0	1	2	3
Number of audio clips/pieces	195	151	41	51
Number of goats/heads	52	41	8	15

**Table 2:** Number of data segmentation segments.

Segmentation Duration	Number of Lung Sounds				
	Normal	Rhonchi	Tachypnea	Noise	Total
10 s	1824	1267	408	674	4173

**Table 3:** Comparison of Model Performance under Different Cropping Durations.

Segmentation Duration	Total Number of Sounds	Accuracy %
6 s	5263	82.67
7 s	4636	85.01
8 s	4173	86.12
9 s	3822	87.01
10 s	4173	87.56
11 s	3822	86.57
12 s	3481	86.31

**Table 4:** Comparison of Results Before and After Data Augmentation.

Method	Accuracy %	Se %	Sp %	Score %
No augmentation	87.56	86.01	88.56	87.28
SMOTE	88.21	86.99	89.14	88.07

**Table 5:** Performance Comparison of Different Feature.

Features	Accuracy %	Se %	Sp %	Score %
MFCC	80.54	78.92	84.61	79.73
CQT	82.51	80.19	86.27	83.23
STFT	84.36	79.69	87.77	83.73
Mel-spectrogram	85.13	82.89	87.32	85.11
Gammatonegram	88.21	86.99	89.14	88.07

**Table 6:** Performance comparison between AAF-SwinT and different models.

Model	Accuracy %	Se %	Sp %	Score %
MobileViT	83.45 (±3.82)	79.99 (±4.05)	88.83 (±3.51)	84.41 (±3.90)
DaViT	85.25 (±2.71)	83.11 (±2.93)	89.02 (±2.48)	86.11 (±2.80)
DeiT	82.23 (±2.77)	79.19 (±2.98)	88.27 (±2.52)	83.73 (±2.85)
ViT	83.29 (±3.02)	84.14 (±3.20)	83.96 (±3.10)	84.05 (±3.15)
Swin Transformer	85.53 (±1.98)	84.76 (±2.15)	87.77 (±1.82)	86.26 (±2.03)
AAF-SwinT	88.21 (±1.91)	86.99 (±2.08)	89.14 (±1.75)	88.07 (±1.95)

**Table 7:** Comparison of attention computation performance across different dimensions.

Attention Strategy	Accuracy %	Se %	Sp %	Score %
MSA	86.53	84.76	87.77	86.26
TA	86.12	84.93	88.64	86.79
FA	86.05	85.68	87.41	86.55
ADA	88.21	86.99	89.14	88.07

**Table 8:** Ablation experiments.

Model Configuration	Accuracy %	Se %	Sp %	Score %	Flops/G	Params/M
base	85.53	84.76	87.77	86.26	4.37	27.50
base + ADA	85.95	85.47	87.87	87.07	4.71	29.66
base + ASAP	86.09	85.02	88.56	86.79	4.25	26.34
base + ADA + ASAP	86.64	85.83	88.99	87.41	4.59	28.5
base + ADA + FAM	85.61	85.25	87.35	86.3	5.77	75
base + ADA + ASAP + FAM	88.21	86.99	89.14	88.07	5.64	73.84

## Data Availability

The dataset is not available temporarily because of confidentiality requirements of commercial goat farm data and the delayed publicity requirements of the funded projects.
